# 三代EGFR-TKI在*EGFR*突变NSCLC治疗中应用的专家共识（2022年版）

**DOI:** 10.3779/j.issn.1009-3419.2022.101.47

**Published:** 2022-09-20

**Authors:** 中国临床肿瘤学会（CSCO）非小细胞肺癌专家委员会, 抗肿瘤药物安全管理专家委员会

**Keywords:** 肺肿瘤, 受体蛋白酪氨酸激酶, ErbB受体, 共识, Lung neoplasms, Receptor protein-tyrosine kinases, ErbB receptors, Consensus

原发性支气管肺癌（以下简称“肺癌”）是我国发病率和死亡率最高的恶性肿瘤，2016年我国肺癌新发病例人数约为82.8万，死亡病例人数约为65.7万^[1]^，而2022年预计新发病例人数约为87万，死亡病例人数约为76万^[2]^。非小细胞肺癌（non-small cell lung cancer, NSCLC）是肺癌中最常见的病理类型，表皮生长因子受体（epidermal growth factor receptor, *EGFR*）基因突变是NSCLC最常见的驱动基因突变。在中国NSCLC人群中，*EGFR*突变比例为28.2%^[3]^，在肺腺癌中，该比例可达50.2%^[4]^。一代/二代EGFR酪氨酸激酶抑制剂（tyrosine kinase inhibitor, TKI）用于*EGFR*经典突变（*EGFR*外显子19缺失突变或外显子21 L858R点突变）晚期NSCLC患者的一线治疗，可使患者的中位无进展生存期（progression-free survival, PFS）提升至9.2个月-14.7个月^[5-13]^，显著优于化疗，但不可避免地出现疾病进展，其中以继发性*EGFR* T790M突变最为常见^[14]^。三代EGFR-TKI可有效抑制*EGFR* T790M突变^[15-17]^，已成为一代/二代EGFR-TKI治疗进展后伴*EGFR* T790M突变患者的标准治疗；同时，由于三代EGFR-TKI一线治疗较一代EGFR-TKI显著延长中位PFS^[18-21]^并带来总生存（overall survival, OS）^[22]^获益，也使其成为了*EGFR*突变晚期NSCLC患者的一线标准治疗。除此之外，三代EGFR-TKI在*EGFR*突变可手术NSCLC、*EGFR*外显子20插入突变NSCLC等患者中的探索和应用也日益广泛，但目前我国尚无针对三代EGFR-TKI临床应用的专家共识。因此，有必要对三代EGFR-TKI的循证医学证据进行总结，为各级临床医师提供规范化的用药指导建议。

本文中采用的共识等级及说明如表 1所示。

**表 1:** 本共识采用的共识等级及说明

共识等级	定义
1级	专家组一致推荐
2A级	大部分专家推荐，有个别争议
2B级	大部分专家推荐，有较多争议
3级	专家组存在较多争议

## 1 三代EGFR-TKI的研发背景和分子结构

为抑制肿瘤细胞生长，EGFR-TKI类药物通过占据EGFR激酶域三磷酸腺苷（adenosine triphosphate, ATP）结合位点，阻断下游生长信号的传递^[23]^。一代EGFR-TKI以喹唑啉作为母环，通过形成氢键与EGFR ATP结合口袋进行可逆性结合^[24]^，当出现*EGFR* T790M突变时，由于受到空间位阻^[25]^和ATP亲和力增加^[26]^的影响，将导致一代EGFR-TKI耐药。二代EGFR-TKI仍以喹唑啉作为母环，通过引入迈克尔加成受体丙烯酰胺，与EGFR激酶域半胱氨酸残基形成不可逆共价结合^[27, 28]^。由于剂量限制性毒性（dose-limiting toxicity, DLT）的影响，二代EGFR-TKI无法增加药物剂量至有效抑制*EGFR* T790M突变^[29]^，会出现继发性*EGFR* T790M突变导致的耐药^[30]^。

WZ4002是首个被报道的三代EGFR-TKI分子，在保留丙烯酰胺的同时，引入嘧啶作为母环，最终显示出针对*EGFR* T790M突变较强的抗肿瘤活性，同时对野生型EGFR抑制作用弱^[24]^。

奥希替尼（AZD9291）是首个成功上市的三代EGFR-TKI，它通过嘧啶母环与EGFR ATP结合口袋铰链区Met793形成氢键，并通过丙烯酰胺与*EGFR* Cys797形成共价键。临床前数据^[32]^显示，奥希替尼对*EGFR* T790M突变显示出较高的抑制活性和较高的选择性^[31]^，并可分布于脑组织中。值得注意的是，奥希替尼有两种主要代谢产物，分别为AZ5104和AZ7550，其中AZ7550与药物原型的活性、选择性相似，而AZ5104对突变型和野生型*EGFR*均具有更强的抑制活性，使其选择性有所降低^[31]^。由于AZ5104为奥希替尼吲哚环去甲基化反应产生，部分新的三代EGFR-TKI尝试对吲哚环上的甲基结构进行优化，以避免产生非选择性代谢产物，如阿美替尼将吲哚环上的甲基替换为环丙基^[33]^，奥瑞替尼则将甲基吲哚替换为6, 7, 8, 9-四氢吡咯并[1, 2-a]吲哚^[34]^，以期在保证抗肿瘤活性的同时，减少因非选择性代谢产物带来的野生型EGFR相关不良反应^[33, 34]^。伏美替尼则引入三氟乙氧基吡啶结构替代奥希替尼的甲氧基苯结构，对多种*EGFR*突变均表现出抑制活性^[35]^。尽管未对吲哚环进行改造，伏美替尼主要活性代谢产物仅有非吲哚环N-去甲基化产生的AST5902，其抗肿瘤活性与选择性均与药物原型相似^[36]^。

总之，不同的三代EGFR-TKI在分子结构上并不相同，而这些分子结构的差异也决定了药物特性的差异。对三代EGFR-TKI分子结构的探讨，有助于理解其临床数据，以更好地指导临床实践。

## 2 *EGFR*相关检测

**共识1：对于完全切除肿瘤原发灶-淋巴结-转移（tumor-node-metastasis, TNM）病理分期Ⅱ期-ⅢA期、术后复发、局部晚期或晚期肺腺癌、含有腺癌成分的NSCLC或非小细胞肺癌非特指型（non-small cell carcinoma not otherwise specified, NSCC-NOS）患者，推荐常规开展*EGFR*突变检测；既往接受一代/二代EGFR-TKI治疗后疾病进展的局部晚期或转移性NSCLC患者，应再次开展基因检测以明确耐药机制（包含*EGFR* T790M突变）。（共识等级：1级）**

*EGFR*常见突变位点发生在外显子18-21上，其中最常见的是外显子19缺失突变和外显子21 L858R点突变，这两种经典突变合计占85%-90%^[37]^。此外，外显子20插入突变占1%-5%^[38-42]^，一代/二代EGFR-TKI治疗后耐药的NSCLC患者中T790M的突变率为50%-60%^[37]^。FLAURA（奥希替尼）^[18]^、AENEAS（阿美替尼）^[19]^、FURLONG（伏美替尼）^[20]^等研究证实局部晚期或转移性NSCLC患者进行*EGFR*突变检测并对突变患者（外显子19缺失突变或外显子21 L858R点突变）一线给予三代EGFR-TKI治疗，中位PFS均超过18个月。ADAURA研究^[43]^证实术后病理确诊IB期-ⅢA期[第7版美国癌症联合委员会（American Joint Committee on Cancer, AJCC）分期]的*EGFR*突变（外显子19缺失突变或外显子21 L858R点突变）NSCLC可从奥希替尼辅助治疗中获益，因此建议在对术后标本常规进行组织病理学诊断时，同步进行*EGFR*基因突变检测。阴性的基因检测结果应谨慎对待，必要时可重复检测。三代EGFR-TKI已经成为一代/二代EGFR-TKI治疗失败后T790M突变NSCLC的标准治疗，因此建议对一代/二代EGFR-TKI治疗进展的NSCLC患者应再次开展基因检测以明确耐药机制（包含*EGFR* T790M突变）^[15-17]^。一些*EGFR*非经典突变（如L861Q、G719X、S768I、外显子20插入突变等）也应常规进行检测^[44-48]^。*EGFR*突变的检测方法主要包括：扩增受阻突变系统法、高通量测序等；对于组织不可获取或组织样本不足的患者，利用液体标本进行基因检测可以作为一种补充手段；液体活检标本包括：血清或血浆循环肿瘤DNA（circulating tumor DNA, ctDNA）、脑脊液和胸腔积液等。

## 3 *EGFR*经典突变晚期NSCLC的一线治疗

**共识2：对于*EGFR*经典突变晚期NSCLC患者，优先推荐三代EGFR-TKI奥希替尼、阿美替尼或伏美替尼单药作为一线治疗。（排名不分先后，推荐级别相同；下同）（共识等级：1级）**

一代EGFR-TKI的问世，使得*EGFR*经典突变（外显子19缺失突变或外显子21 L858R点突变）晚期NSCLC患者的中位PFS可以达到9.2个月-13.1个月，较既往标准化疗显著延长^[6-11]^。二代EGFR-TKI较一代EGFR-TKI疗效更佳，中位PFS可达11.0个月-14.7个月，但不良事件发生率也明显增加^[13, 47]^。三代EGFR-TKI一线治疗*EGFR*经典突变晚期NSCLC的研究结果显示：三代EGFR-TKI较一代EGFR-TKI具有更好的有效性和更优的安全性，中位PFS达到17.8个月-20.8个月，≥3级治疗相关的不良事件发生率为11%-25%^[18-22]^。

目前已经公布了晚期一线治疗Ⅲ期临床研究^[18-22]^结果的三代EGFR-TKI包括奥希替尼、阿美替尼及伏美替尼，并且这三款药物均已在中国获批晚期一线治疗的适应证，三代EGFR-TKI的上市为*EGFR*经典突变晚期NSCLC患者提供了更好的治疗选择。

FLAURA研究^[18]^全球人群结果显示：奥希替尼组较一代EGFR-TKI（吉非替尼或厄洛替尼）组显著延长中位PFS（18.9个月 *vs* 10.2个月，HR=0.46，*P* < 0.001）。数据截止至2019年6月25日，OS成熟度为58%，奥希替尼组较对照组显著延长中位OS（38.6个月 *vs* 31.8个月，HR=0.80，*P*=0.046）^[22]^。在安全性方面，奥希替尼组≥3级治疗相关的不良事件的发生率低于对照组，两组分别为18%和28%，奥希替尼组常见的治疗相关不良事件（发生率≥20%）包括：皮疹（54%）、腹泻（49%）、皮肤干燥（33%）、甲沟炎（33%）、口腔炎（25%）^[18]^，详见不良反应管理章节。

FLAURA研究^[21]^中国人群队列结果显示：奥希替尼组较吉非替尼组显著延长中位PFS（17.8个月 *vs* 9.8个月，HR=0.56，*P*=0.007）。数据截止至2019年6月25日，OS成熟度为65%，奥希替尼组与对照组相比未显著延长中位OS（33.1个月 *vs* 25.7个月，HR=0.85，*P*=0.442）。在安全性方面，奥希替尼组≥3级治疗相关的不良事件的发生率高于对照组，两组分别为25%和15%，奥希替尼组常见的治疗相关不良事件（发生率≥20%）包括：白细胞计数下降（33.8%）、血小板计数下降（25.4%）、中性粒细胞计数下降（21.1%），详见不良反应管理章节。

AENEAS研究^[19]^结果显示：阿美替尼组较吉非替尼组显著延长中位PFS（19.3个月 *vs* 9.9个月，HR=0.46，*P* < 0.000, 1）。数据截止至2021年1月15日，OS成熟度为29%。在安全性方面，阿美替尼组≥3级治疗相关不良事件的发生率低于对照组，两组分别为20.1%和26.5%，阿美替尼组常见治疗相关不良事件（发生率≥20%）包括：血肌酸磷酸激酶（creatine phosphokinase, CPK）升高（34.1%）、谷丙转氨酶升高（28.0%）、谷草转氨酶升高（27.6%）、白细胞计数下降（20.6%）、皮疹（20.6%），详见不良反应管理章节。

FURLONG研究^[20]^结果显示：伏美替尼组较吉非替尼组显著延长中位PFS（20.8个月 *vs* 11.1个月，HR=0.44，*P* < 0.000, 1）。数据截止至2021年9月15日，OS成熟度为32%。在安全性方面，伏美替尼组≥3级治疗相关不良事件的发生率低于对照组，两组分别为11%和18%，伏美替尼组常见的治疗相关不良事件（发生率≥20%）包括：谷丙转氨酶升高（28%）、谷草转氨酶升高（25%）、腹泻（25%），详见不良反应管理章节。

目前，更多新的三代EGFR-TKI正在开展用于晚期一线治疗的临床研究，期待产生更多的循证医学证据，进一步验证三代EGFR-TKI单药是*EGFR*经典突变晚期NSCLC患者的一线优选。

## 4 *EGFR* T790M突变晚期NSCLC的二线/后线治疗

**共识3：对于一代/二代EGFR-TKI治疗后存在*EGFR* T790M突变晚期NSCLC患者，推荐三代EGFR-TKI奥希替尼、阿美替尼或伏美替尼单药作为二线/后线治疗。（共识等级：1级）**

*EGFR*经典突变晚期NSCLC患者接受一代/二代EGFR-TKI治疗后，约50%患者会出现T790M耐药突变^[14, 49]^。三代EGFR-TKI保留了二代EGFR-TKI的丙烯酰胺，将喹唑啉母环更换为嘧啶母环，并对侧链进行分子结构的改造，从而成功克服T790M耐药突变^[15-17, 33, 35, 50-53]^。目前中国已有三种三代EGFR-TKI获批用于治疗*EGFR* T790M突变晚期NSCLC，按照获批顺序依次为奥希替尼、阿美替尼以及伏美替尼。

奥希替尼在剂量爬坡研究中，从20 mg/d升至240 mg/d，未观察到DLT和最大耐受剂量（maximum tolerable dose, MTD）^[51]^。在AURA2和AURA3研究^[15, 52]^中，奥希替尼的客观缓解率（objective response rate, ORR）达到70%-71%，PFS达到9.9个月-10.1个月。奥希替尼在亚太人群中进行的AURA17研究^[53]^结果显示：主要研究终点ORR达到63%，亚组分析发现：合并*EGFR*外显子19缺失突变人群的ORR为64%，合并*EGFR*外显子21 L858R点突变人群的ORR为59%；次要研究终点中位PFS为9.7个月。在安全性方面，奥希替尼≥3级治疗相关不良事件的发生率为11%，常见的不良事件（发生率≥20%）包括：腹泻（35%）、皮疹（27%）、咳嗽（22%）、白细胞减少（20%），详见不良反应管理章节。

阿美替尼在剂量爬坡研究中，从55 mg/d升至260 mg/d，在220 mg/d剂量组和260 mg/d剂量组各观察到1例DLT，未观察到MTD^[33]^。在Ⅱ期研究（APOLLO）^[16]^中，主要研究终点ORR达到68.9%，亚组分析发现：合并*EGFR*外显子19缺失突变人群的ORR为72.2%，合并*EGFR*外显子21 L858R点突变人群的ORR为63.5%；次要研究终点中位PFS为12.4个月。在安全性方面，阿美替尼≥3级治疗相关不良事件的发生率为16.4%，常见的不良事件（发生率≥20%）包括：血CPK升高（20.9%）、上呼吸道感染（20.5%），详见不良反应管理章节。

伏美替尼在剂量爬坡研究^[35]^中，从20 mg/d升至240 mg/d，未观察到DLT和MTD。在Ⅱb期研究（ALSC003）^[17]^中，主要研究终点ORR达到74%，亚组分析发现：合并*EGFR*外显子19缺失突变人群的ORR为76.5%，合并*EGFR*外显子21 L858R点突变人群的ORR为71.4%；次要研究终点中位PFS为9.6个月。在安全性方面，伏美替尼≥3级治疗相关不良事件的发生率为11%，未观察到发生率≥20%的不良事件，详见不良反应管理章节。

此外，还有多款新的三代EGFR-TKI已经陆续公布了用于*EGFR* T790M突变晚期NSCLC患者的Ⅱ期研究结果（包括：贝福替尼、瑞齐替尼、奥瑞替尼和Limertinib等）^[54-57]^，均显示出一定疗效，更多新的三代EGFR-TKI正在研发中^[58]^，将为患者提供更多潜在的治疗选择。

在奥希替尼的AURA研究^[59]^和伏美替尼的两项Ⅱ期研究^[17, 35]^中，分别纳入了7例和10例原发性T790M突变晚期NSCLC患者（奥希替尼AURA研究中的7例患者中3例患者为亚洲人群、4例患者为高加索人群；伏美替尼的两项Ⅱ期研究中的10例患者均为中国人群），ORR分别为85.7%和90%^[17, 35, 59]^，考虑三代EGFR-TKI对原发性T790M突变晚期NSCLC患者有效，可尝试应用，并鼓励开展相关临床研究。

## 5 可手术*EGFR*经典突变NSCLC围手术期治疗

**共识4：对于完全切除TNM病理分期为Ⅱ期-ⅢA期*EGFR*经典突变NSCLC患者辅助化疗后，推荐三代EGFR-TKI单药作为辅助治疗（目前奥希替尼已获批该适应证）。（共识等级：2A级）**

ADAURA研究^[43]^是一项随机、双盲、Ⅲ期临床试验，共纳入了682例IB期-ⅢA期、*EGFR*经典突变、肿瘤完全切除术后的NSCLC患者，按1:1随机分为两组，分别接受奥希替尼或安慰剂3年辅助治疗。主要终点是Ⅱ期-ⅢA期患者的无病生存期（disease free survival, DFS）；次要终点包括全人群（IB期-ⅢA期）的DFS、OS和安全性。2020年公布的结果显示，24个月时，Ⅱ期-ⅢA期NSCLC患者中，奥希替尼组和安慰剂组的DFS率分别为90%和44%（HR=0.17, *P* < 0.001）。全人群中，奥希替尼组和安慰剂组的DFS率分别为89%和52%（HR=0.20, *P* < 0.001）。OS数据尚未成熟。患者无论是否接受辅助化疗，均可从三代EGFR-TKI辅助治疗中获益，化疗组HR=0.16（95%CI: 0.10-0.26），未接受化疗组HR=0.23（95%CI: 0.13-0.40）。2022年欧洲肺癌大会（European Lung Cancer Congress, ELCC）公布了中国队列结果^[60]^，中国人群（*n*=159）Ⅱ期-ⅢA期NSCLC患者中，中位DFS在奥希替尼组未达到，安慰剂组为18.3个月，HR=0.16（95%CI: 0.08-0.31）。IB期-ⅢA期患者的DFS在奥希替尼组未达到，安慰剂组为24.9个月，HR=0.18（95%CI: 0.10-0.33）。在中国队列的所有预设亚组中均观察到奥希替尼与安慰剂相比存在DFS的获益。三代EGFR-TKI用于术后辅助治疗的多项Ⅲ期注册临床研究^[58]^正在进行中[CTR20220984 (ADAURA2), CTR20202460, CTR20210429 (FORWARD)]。

关于治疗人群，由于ADAURA^[43]^、ADJUVANT^[61]^等研究基于美国癌症联合会（American Joint Committee on Cancer, AJCC）第7版TNM分期评估受试者，入组的ⅢA期患者，相当于目前AJCC第8版ⅢB期患者，因此对于AJCC第8版TNM分期的Ⅱ期-ⅢB期患者，ADAURA^[43]^、ADJUVANT^[61]^、EVAN^[62]^、EVIDENCE^[63]^等研究显示出EGFR-TKI辅助治疗的获益。关于IB期患者术后是否接受辅助靶向治疗，目前尚存在一定争议。目前公布结果的Ⅲ期临床试验中，除ADAURA包含IB期患者之外，其他研究均只纳入了Ⅱ期及更高分期的患者。ADAURA研究^[43]^中IB期患者奥希替尼组对比安慰剂组的DFS HR=0.39（95%CI: 0.18-0.76, *P* < 0.001）。

关于辅助治疗时长的问题，ADJUVANT、EVAN和EVIDENCE研究中术后辅助靶向治疗时长为2年，ADAURA研究设计中奥希替尼的辅助治疗时间为3年^[43, 62-64]^。因此目前认为术后1年的辅助靶向治疗时长不够，术后辅助靶向治疗的时长至少为2年。

三代EGFR-TKI用于新辅助治疗多项临床研究正在进行中，包括奥希替尼用于新辅助治疗的NeoADAURA研究和NEOS研究^[65]^，阿美替尼用于围术期Ⅲ期研究（CTR20201857, ChiCTR2100042856）正在进行中，伏美替尼目前布局了围术期Ⅱ期研究（FRONT）。

## 6 *EGFR*外显子20插入突变NSCLC的治疗

**共识5：对于*EGFR*外显子20插入突变晚期NSCLC患者标准化疗后，可选择伏美替尼治疗。（共识等级：2A级）**

*EGFR*外显子20插入突变作为*EGFR*突变频率较高的一种非经典突变，在中国人群中占*EGFR*基因突变的2%-5%，占NSCLC的0.3%-2.9%^[66]^。*EGFR*外显子20插入突变异质性高，目前已报道了122种突变亚型^[67]^。

*EGFR*外显子20插入突变晚期NSCLC患者的一线治疗，国内外指南仍然推荐参照无驱动基因突变NSCLC的治疗方案，在含铂双药化疗失败后，推荐Amivantamab或Mobocertinib^[68, 69]^，但这两款新药暂未在中国获批上市，尚不可及。在两项中国回顾性研究^[70, 71]^中发现，针对该类患者晚期一线化疗的疗效不佳，ORR为19.2%-41.6%，中位PFS为3.0个月-6.4个月；二线化疗ORR仅为17.6%，中位PFS为4.0个月。接受一代/二代EGFR-TKI作为晚期一线治疗，ORR仅为0%-8.7%，中位PFS仅为2.0个月-2.7个月^[46, 70]^。

三代EGFR-TKI奥希替尼在此类人群中的疗效不甚理想，两项前瞻性研究^[72, 73]^结果显示：奥希替尼80 mg/d的剂量未带来肿瘤缓解，ORR均为0，中位PFS为3.5个月-3.8个月。两项回顾性研究^[74, 75]^结果显示：奥希替尼80/160 mg/d ORR仅为5%-6.5%，中位PFS为2.3个月-3.6个月。在另外两项前瞻性研究^[76, 77]^中，奥希替尼剂量提升至160 mg/d，ORR达到24%-28%，中位PFS达到6.8个月-9.6个月，但不良事件发生率较高，常见的不良事件包括腹泻（72%-76%）、乏力（44%-67%）、血小板计数下降（20%-67%）等。

阿美替尼尚无前瞻性研究数据公布，目前仅发表了1篇病例报道：在1例*EGFR*外显子20插入突变（p.A763_Y764insFQEA）晚期NSCLC患者中，接受以阿美替尼为核心的综合治疗，疗效评价为部分缓解（partial response, PR），PFS达到10个月^[78]^。

伏美替尼公布的临床前体外研究结果显示：伏美替尼可有效抑制多种常见的*EGFR*外显子20插入突变亚型。临床前体内研究结果显示：伏美替尼50 mg/kg/d可有效抑制携带*EGFR*外显子20插入突变（p.N771_H773dup）的移植瘤模型。FAVOUR研究^[79]^初步结果显示：伏美替尼240 mg/d一线治疗*EGFR*外显子20插入突变NSCLC患者ORR达到60%，疾病控制率（disease control rate, DCR）达到100%，药物中位暴露时间4.1个月时，所有患者均仍在接受治疗，未出现疾病进展或死亡；且安全性良好，未出现≥3级不良事件，未观察到因不良事件导致的药物减量或研究终止。两项真实世界回顾性研究^[80, 81]^结果显示：伏美替尼160 mg/d ORR可达到53.3%-55.6%，安全性良好，均未观察到≥3级不良事件发生。此外，两篇病例报道^[82, 83]^进一步证实了高剂量伏美替尼的疗效和安全性。

目前，更多新的三代EGFR-TKI也在开展针对*EGFR*外显子20插入突变晚期NSCLC的研究，包括：BEBT-109、JFAN-1001、PLB1004等^[58]^，不同的三代EGFR-TKI对*EGFR*外显子20插入突变晚期NSCLC的疗效不同，循证医学证据等级不同，仍需进一步探索，鼓励临床医生开展相关的临床研究。

## 7 *EGFR*经典突变NSCLC伴中枢神经系统转移的治疗

**共识6：对于*EGFR*经典突变NSCLC伴中枢神经系统（central nervous system, CNS）转移患者，靶向药物优先选择三代EGFR-TKI（奥希替尼、阿美替尼或伏美替尼）。（共识等级：1级）**

CNS转移是晚期NSCLC最常见的转移部位之一。*EGFR*突变NSCLC初诊时约25%的患者合并CNS转移，诊断3年后晚期NSCLC发生CNS转移的比例超过45%^[84, 85]^。NSCLC伴CNS转移患者预后差，自然生存仅为1个月-2个月^[86]^。对于有症状、颅内单个病灶或有其他局部治疗指征的*EGFR*突变NSCLC伴CNS转移患者，应在全身治疗基础上积极进行局部治疗，包括外科手术、全脑放疗、立体定向放疗等；对于无症状的*EGFR*突变NSCLC CNS转移患者，可选用能更好穿透血脑屏障的三代EGFR-TKI进行全身治疗。

相比一代EGFR-TKI，三代EGFR-TKI穿透血脑屏障的能力更强，药物入脑浓度更高。有动物实验^[32]^显示，奥希替尼、吉非替尼和阿法替尼的药峰浓度（maximum concentration, Cmax）脑组织/血浆比（brain/plasma Cmax ratio）分别为3.41、0.21和 < 0.36。临床前研究^[35, 87]^结果显示，阿美替尼和伏美替尼同样可以穿透血脑屏障，在脑组织中分布较好。

多项临床研究结果显示三代EGFR-TKI用于*EGFR*经典突变NSCLC伴CNS转移患者具有良好的有效性和安全性。FLAURA研究^[18]^显示，奥希替尼一线治疗*EGFR*经典突变NSCLC伴CNS转移患者的中位PFS获益明显优于一代EGFR-TKI（15.2个月 *vs* 9.6个月，HR=0.47，*P* < 0.001）。AURA3研究^[88]^显示，对于EGFR-TKI治疗进展后伴*EGFR* T790M突变的NSCLC伴CNS转移患者，奥希替尼组对比化疗组可明显提高颅内中位PFS（11.7个月 *vs* 5.6个月，HR=0.32，*P*=0.004）和颅内ORR（70% *vs* 31%, OR=5.13, *P*=0.015）。AENEAS研究^[19]^CNS转移亚组中，阿美替尼组和吉非替尼组的中位PFS分别为15.3个月和8.2个月（HR=0.38, *P* < 0.000, 1）。APOLLO研究^[16]^提示，阿美替尼治疗一代/二代EGFR-TKI治疗进展后*EGFR* T790M突变NSCLC伴CNS转移患者的CNS ORR为60.9%，CNS DCR为91.3%，中位PFS为11.8个月。伏美替尼一线治疗*EGFR*经典突变NSCLC的Ⅲ期FURLONG研究中，关于CNS转移亚组，伏美替尼组和吉非替尼组的中位PFS分别为18.0个月和11.2个月（HR=0.514, *P*=0.002, 8）^[20]^；CNS PFS两组分别为20.8个月和9.8个月（HR=0.40, *P*=0.001, 1）^[89]^。另一项伏美替尼用于EGFR-TKI治疗进展后伴*EGFR* T790M突变NSCLC的ⅡB期研究^[17]^提示，伏美替尼治疗CNS转移患者，CNS ORR达66%，CNS DCR达100%，CNS PFS为11.6个月。

FURLONG^[89]^、FLAURA研究事后分析^[18]^和ADAURA研究^[43]^均提示，无论是晚期一线还是辅助治疗阶段，对于尚无CNS转移病灶的*EGFR*突变NSCLC患者，伏美替尼和奥希替尼可降低CNS转移风险。FURLONG研究^[90]^对基线无CNS转移患者的事后分析显示，在随访期间伏美替尼组没有新发CNS转移，而吉非替尼组中有8例出现CNS转移，提示伏美替尼一线治疗可预防或推迟CNS转移的发生。FLAURA研究^[18]^中，奥希替尼组和一代EGFR-TKI组新发CNS转移的比例分别为3%（7/226）和7%（15/214）。ADAURA研究^[43, 91]^中，IB期-ⅢA期*EGFR*经典突变、完全切除术（R0切除）后的NSCLC患者接受奥希替尼辅助治疗，相比安慰剂组可明显降低患者术后CNS复发率（分别为 < 1%和10%）。

目前三代EGFR-TKI尚无针对脑转移的前瞻性大型临床研究，现有数据大多来自亚组分析，证据等级有限。建议开展三代EGFR-TKI针对*EGFR*突变NSCLC脑转移和脑膜转移患者的前瞻性大型临床研究，也可探索加量、联合治疗的获益情况。

一项前瞻性临床研究^[92]^纳入11例*EGFR*突变NSCLC脑转移患者，在应用奥希替尼80 mg/d出现CNS进展后，增加剂量至160 mg/d，仍可使部分患者达到颅内疾病控制，颅内ORR可达54%，中位颅内PFS为4.3个月。另一项研究^[93]^中，奥希替尼160 mg/d治疗*EGFR*突变NSCLC脑转移和脑膜转移（各40例），DCR分别为77.5%和92.5%，中位OS分别为16.9个月和13.3个月。伏美替尼治疗*EGFR* T790M突变局部晚期或转移性NSCLC的Ⅰ期-Ⅱ期剂量扩展研究（NCT03127449）的CNS转移人群数据结果^[94]^显示，80 mg/d伏美替尼的CNS ORR达到60.0%，而160 mg/d伏美替尼的CNS ORR达到84.6%，CNS DCR达100%，颅内中位PFS为19.3个月，加量对疗效提升具有临床意义。目前阿美替尼推荐剂量为110 mg/d，阿美替尼Ⅰ期临床试验^[33]^显示，阿美替尼剂量递增至220 mg/d时，安全可耐受。

一项评估奥希替尼联合贝伐珠单抗用于一线治疗的Ⅰ期/Ⅱ期临床研究^[95]^纳入49例患者，其中6例基线合并脑转移患者ORR达100%。奥希替尼+培美曲塞/铂类一线治疗（FLAURA2研究）（NCT04035486）和拉泽替尼+Amivantamab一线治疗（MARIPOSA研究）（NCT04487080）正在进行中，将为联合化疗或联合靶向治疗对于脑转移人群是否可以带来较多获益提供更多循证医学证据。

## 8 三代EGFR-TKI的联合治疗

**共识7：除临床研究外，不常规推荐三代EGFR-TKI联合抗血管生成药物用于*EGFR*经典突变晚期NSCLC一线治疗，以及一代/二代EGFR-TKI耐药后患者的二线/后线治疗；不常规推荐三代EGFR-TKI联合化疗用于一代/二代EGFR-TKI耐药后患者的二线/后线治疗。（共识等级：2B）**

近年来对EGFR-TKI联合治疗策略如联合化疗或抗血管生成药物等进行了广泛探索，旨在延缓EGFR-TKI耐药，最终延长患者OS。一代EGFR-TKI联合化疗或抗血管生成药物的治疗方案，相比于单药可延长患者PFS^[96-105]^，其中，部分联合化疗的Ⅲ期临床研究^[97, 98]^也显示出显著的OS获益。然而，采用三代EGFR-TKI联合化疗或抗血管生成药物的获益尚未得到前瞻性、Ⅲ期随机对照临床研究的验证。

三代EGFR-TKI单药是一代/二代EGFR-TKI治疗进展后伴*EGFR* T790M突变晚期NSCLC人群的标准治疗。有研究^[106]^在这类人群中探索了三代EGFR-TKI联合治疗模式：JVDL研究（1期，共25例患者）显示，奥希替尼联合雷莫芦单抗治疗的中位PFS为11.0个月，24个月OS率为52%，与历史数据对照无优势；WJOG8715L研究（2期，*n*=81）^[107]^显示，奥希替尼联合贝伐珠单抗的中位PFS为9.4个月，不优于奥希替尼单药治疗的13.5个月（HR=1.44, *P*=0.20）；BOOSTER研究（2期，*n*=155）^[108]^显示，奥希替尼联合贝伐珠单抗组的中位PFS为15.4个月，与对照组（奥希替尼单药）的中位PFS 12.3个月相仿（HR=0.95, *P*=0.83）；NEJ032A研究（2期，*n*=62）^[109]^评估了奥希替尼联合卡铂/培美曲塞对比奥希替尼单药二线治疗的有效性，两组PFS分别为15.8个月和14.6个月（HR=1.09, *P*=0.83）。以上结果尚不支持在一代/二代EGFR-TKI治疗后*EGFR* T790M耐药突变患者中，使用三代EGFR-TKI联合化疗或抗血管生成药物治疗策略。

三代EGFR-TKI联合抗血管生成药物用于*EGFR*经典突变NSCLC一线治疗是当前的探索热点之一：一项纳入49例患者的1期/2期单臂研究^[95]^显示，奥希替尼联合贝伐珠单抗一线治疗的中位PFS为19个月；AUTOMAN研究^[110]^评估了奥希替尼联合安罗替尼一线治疗的初步有效性，ORR为65.2%；WJOG9717L研究^[111]^对奥希替尼联合贝伐珠单抗和奥希替尼单药一线治疗进行了对比，两组中位PFS相似（22.1个月 *vs* 20.2个月，HR=0.862，*P*=0.213），而不良事件发生率增加。以上研究结果尚不足以支持三代EGFR-TKI联合抗血管生成治疗用于*EGFR*经典突变晚期NSCLC患者的一线治疗。

三代EGFR-TKI一线联合化疗是联合治疗的另一个探索方向。一项小样本2期研究^[112]^纳入67例患者，评估了奥希替尼联合铂类/培美曲塞一线治疗的安全性和有效性，结果显示联合顺铂/培美曲塞或卡铂/培美曲塞的安全性数据相似，24个月PFS率分别为53.2%、86.8%，提示三代EGFR-TKI联合化疗用于*EGFR*经典突变NSCLC的一线治疗模式可能具有更好的前景。

联合治疗模式也在选择性人群中开展了很多探索。一些研究的结果认为疾病进展较快或肿瘤负荷较大的患者可能是联合治疗模式的适用人群。有研究者探索了针对脑转移患者采取联合贝伐珠单抗治疗（详见第7章节：*EGFR*经典突变NSCLC伴脑转移的治疗）；对于L858R突变人群采取联合贝伐珠单抗治疗（NCT04988607）；对于非经典突变人群采取联合贝伐珠单抗治疗（NCT04974879）等。随着分子检测技术的发展和应用，基于ctDNA检测结果采取联合治疗已成为新的探索方向，一些三代EGFR-TKI联合治疗的研究正在进行[NCT05334277 (FOCUS-C), NCT04410796, NCT04769388 (FLAME), NCT04500704 (ACROSS1), NCT04500717 (ACROSS2)]。

三代EGFR-TKI联合治疗尚有多项Ⅲ期研究值得关注，如一线联合化疗[NCT04035486 (FLAURA2), NCT04923906]、一线联合抗血管生成治疗（NCT04181060）、一线联合EGFR/间质表皮转化因子（mesenchymal-epithelial transition, MET）双抗治疗（NCT04487080）、一线联合EGFR/人表皮生长因子受体3（human epidermal growth factor receptor 3, HER3）双抗治疗（NCT05020769）等，这些研究数据的公布将有助于进一步明确三代EGFR-TKI多种联合治疗模式的有效性和安全性。

综上所述，现有证据尚不足以支持三代EGFR-TKI与化疗或抗血管生成治疗联合用于晚期*EGFR*经典突变患者的一线治疗或用于一代/二代EGFR-TKI治疗失败且T790M突变患者。期待多项三代EGFR-TKI与其他药物联合一线治疗晚期*EGFR*经典突变NSCLC研究结果的揭晓，以进一步明确联合治疗的价值，为*EGFR*突变NSCLC精准化、个体化治疗方案的制定提供循证医学证据。

## 9 三代EGFR-TKI的不良反应及管理

**共识8：不同三代EGFR-TKI不良反应谱总体相似，包括皮疹、腹泻、肝功能异常、血液系统毒性等，在发生频率和严重程度上存在差异。（共识等级：1级）**

**共识9：对于某种三代EGFR-TKI不良反应不能耐受的患者，可换用另一种三代EGFR-TKI。（共识等级：3级）**

与一代/二代EGFR-TKI相比，三代EGFR-TKI不良反应谱总体相似，但不同三代EGFR-TKI之间也存在一定差异。根据现有报道，三代EGFR-TKI的主要不良反应及其发生率见表 2（注：以下数据源自相关药物中国说明书中对常见不良反应的汇总。如果说明书中对某项不良反应无具体发生率数据呈现，则引用其关键临床研究数据。具体来源请查阅相应参考文献）^[16, 19-22, 43, 52, 53, 113-118]^。

**表 2:** 不同三代EGFR-TKI中不良反应的发生率[%（≥3级，%）]

不良反应	奥希替尼	阿美替尼	伏美替尼
皮疹	45（0.7）^[113]^	23.7（0.4）^[114]^	14.8（0）^[115]^
腹泻	47（1.4）^[113]^	12.5（0.8）^[114]^	15.2（1.1）^[115]^
血液系统毒性			
白细胞减少	65（1.2）^[113]^	16.5（1.0）^[114]^	14.6（0）^[115]^
血小板减少	53（1.2）^[113]^	13.5（0.8）^[114]^	7.4（0.9）^[115]^
贫血	11-38（1-3）^[21-22, 53, 117, 118]^	14.7（0.8）^[114]^	9（0.2）^[115]^
肝功能异常			
转氨酶升高	7-16（1-4.2）^[21-22, 117, 118]^	13.1-29.9（0.4-2.8）^[16, 19]^	21.1-22.0（1.3）^[115]^
GGT升高	9（2.8）^[21]^	8.9（0.9）^[19]^	5.4（1.1）^[115]^
胆红素升高	NR^a^^[22]^	8.4（0）^[19]^	2（1）^[20]^
心脏毒性			
QT间期延长	0.8^b^^[113]^，5-14（1-3）^[21-22, 43, 117, 118]^）	6.1-10.7（0-0.9）^[16, 19]^	12.8（1.3）^[115]^
LVEF下降	1.6-3.2（NR）^[113]^	1.6（NR）^[114]^	0.4（0）^[115]^
血CPK升高	1（1）^[52]^	26.8（6.4）^[114]^	5.2（0.2）^[115]^
间质性肺疾病	3.7（1.1）^[113]^	0.4（0）^[114]^	0.4（0.2）^[115]^
CPK：肌酸磷酸激酶；EGFR-TKI：表皮生长因子受体酪氨酸激酶抑制剂；GGT：*γ*-谷氨酰转移酶；LVEF：左心室射血分数；NR：未报告。 ^a^仅一项研究中报告了1例因胆红素升高导致的严重不良事件^[22]^；^b^说明书中报告的发生率为QTcF延长 > 500 ms的发生率。			

如表 2所述，几种三代EGFR-TKI常见不良反应的发生率和严重程度有所不同。例如，奥希替尼皮疹、腹泻、白细胞减少、血小板减少等类型的不良事件发生率相对较高，而阿美替尼血CPK升高的发生率相对较高。临床实践中可针对患者的具体情况，结合不同三代EGFR-TKI不良反应发生率及严重程度的差异，制定个体化的治疗方案。

针对以上不良反应的预防和处理，可参考国内发布的《EGFR-TKI不良反应管理专家共识》^[119]^和相应药物说明书^[113-115]^进行。一些个案报道提示，在部分严重不良反应恢复后，在患者充分知情及密切监测的基础上，可考虑原三代EGFR-TKI药物的再挑战应用，如史蒂文斯-约翰逊综合征恢复后在口服激素治疗（逐渐减停）的基础上再挑战^[120]^，间质性肺疾病恢复后在口服激素治疗的基础上低剂量再挑战等^[121]^。考虑到目前有多个三代EGFR-TKI可供选择，且不同三代EGFR-TKI在不良事件的发生率或严重程度上存在差异，不同三代药物之间的替换成为一种探索方向。目前已有一些个案报道，针对某种三代EGFR-TKI不良反应不耐受患者，换用另一种三代EGFR-TKI治疗，总体有效性和耐受性良好^[122-124]^。需要注意的是，目前尚缺乏大型前瞻性临床研究数据，且三代EGFR-TKI不良反应谱总体相似，患者之间也存在个体差异，换药治疗需谨慎开展。

总体而言，三代EGFR-TKI的安全性和耐受性良好，治疗方案可根据患者具体情况和不同药物的安全性特征进行选择。针对不良反应及时开展规范的预防和管理，将有助于进一步提高患者依从性，改善生活质量，最终提升患者的OS。

## 10 三代EGFR-TKI的耐药机制及耐药后治疗探索

尽管三代EGFR-TKI为*EGFR*突变NSCLC患者带来了更多的生存获益，耐药问题仍然无法避免。与一代/二代EGFR-TKI耐药机制相似，目前报道的三代EGFR-TKI的耐药机制主要包括*EGFR*继发性突变、旁路激活、组织学转化等^[17, 125, 126]^。在*EGFR*继发性突变耐药中，最常见的耐药机制为C797X突变，见于奥希替尼（一线/二线治疗后）0%-29%的耐药患者^[125]^、阿美替尼（二线治疗后）21%的耐药患者^[126]^以及伏美替尼（二线治疗后）9%的耐药患者^[17]^。在旁路激活方面，奥希替尼最常见的耐药机制为*MET*基因扩增，占7%-24%^[125]^；阿美替尼最常见为磷脂酰肌醇激酶-3催化亚单位α（phosphatidylinositol-4, 5-bisphosphate 3-kinase, catalytic subunit alpha, *PIK3CA*）基因突变，约占19%^[126]^；伏美替尼最常见为*HER2*基因扩增，约占9%^[17]^。此外，2%-15%接受奥希替尼治疗的患者会出现小细胞肺癌（small cell lung cancer, SCLC）转化^[125]^，7%的患者出现鳞癌转化^[127]^，阿美替尼和伏美替尼则暂未报道组织学转化的发生比例^[17, 126]^，值得进一步探索。

第四代EGFR-TKI为针对*EGFR* C797X继发突变而开发的一类新药，目前尚无药物上市，但部分药物在临床前试验中展现出良好的抗肿瘤活性，并已进入临床阶段，如BBT-176（NCT04820023）、BLU-945（NCT04862780）^[128, 129]^、BLU-701（NCT05153408）^[130]^、BPI-361175（NCT05329298, NCT05393466）等，这些药物的开发将有助于应对*EGFR* C797X突变导致的耐药问题。针对旁路激活导致的耐药，则可联合特定的靶向药物治疗，如MET抑制剂^[131, 132]^、转染重排（rearranged during transfection, RET）TKI^[133]^、间变性淋巴瘤激酶（anaplastic lymphoma kinase, ALK）TKI^[134, 135]^等。针对SCLC转化，则可采取铂/依托泊苷化疗^[136]^，鳞癌转化可采取基于组织学的含铂化疗^[125]^等治疗方式。当耐药机制不明确或治疗方案不可及时，则建议采取含铂化疗，或尝试阿替利珠单抗联合贝伐珠单抗和卡铂/培美曲塞方案^[137, 138]^，或参加临床研究^[125]^。

综上所述，三代EGFR-TKI耐药机制已逐渐明确，一些具有前景的治疗方案已被用于临床实践，或处于进一步开发中。针对不同的耐药机制而采取不同的治疗策略，将推动接受三代EGFR-TKI治疗的患者生存获益最大化。

## 11 总结与展望

NSCLC的治疗已步入精准治疗时代，以EGFR-TKI为代表的分子靶向治疗的问世，彻底改变了既往NSCLC的治疗模式。三代EGFR-TKI的开发，通过引入嘧啶母环和丙烯酰胺结构，成功克服了一代/二代EGFR-TKI应用后出现的*EGFR* T790M耐药突变。不同三代EGFR-TKI的研发思路不尽相同，主要体现在对侧链的改造方面，但都是希望能够通过相关的分子结构优化以提高药物的亲和力、有效性，减少药物的脱靶效应和不良反应。目前在中国批准上市的三种三代EGFR-TKI在*EGFR*突变晚期NSCLC中，均获得了*EGFR*经典突变晚期NSCLC一线治疗和*EGFR* T790M突变晚期NSCLC二线/后线治疗的适应证，因此，三代EGFR-TKI单药治疗在*EGFR*突变晚期NSCLC中地位举足轻重，但对特定人群（如ctDNA未清除、合并共突变等）是否需要进行联合治疗以及最佳的联合治疗模式等问题，仍需进一步明确。目前三代EGFR-TKI的应用和探索已从晚期患者拓展至围手术期患者，三代EGFR-TKI术后辅助治疗的地位明确，新辅助治疗的最佳治疗模式和获益人群正在探索当中。近年来*EGFR*外显子20插入突变成为研发的热门靶点之一，不同三代EGFR-TKI的疗效和循证医学证据等级差异较大，可能是由于各个药物研发思路的不同带来的结果，期待循证医学证据等级更高的临床试验结果公布，支持三代EGFR-TKI在这部分人群的应用。对于*EGFR*突变合并脑转移NSCLC人群，三代EGFR-TKI显示出更好的有效性，未来的探索方向倾向于三代EGFR-TKI加量或者联合治疗等模式。三代EGFR-TKI总体安全性和耐受性良好，但不同的三代EGFR-TKI不良反应数据存在差异，且患者具有个体差异，选择药物时需根据患者的具体情况制定个体化的治疗方案。

三代EGFR-TKI的研发正如火如荼地开展，进一步优化治疗模式是未来探索的方向。本共识将根据临床研究数据的更新、临床检测技术的发展及临床操作实践的进步定期更新，为规范三代EGFR-TKI的应用提供指导和建议，进而推进中国肺癌诊疗技术的发展，更好地造福肺癌患者。

**Table:** 《三代EGFR-TKI在EGFR突变NSCLC治疗中应用的专家共识》专家组成员

	专家姓名	单位
组长	周彩存	同济大学附属上海市肺科医院
	秦叔逵	南京大学医学院附属金陵医院
副组长	王洁	中国医学科学院肿瘤医院
	程颖	吉林省肿瘤医院
参与专家（以姓氏汉语拼音为序，*标注为执笔专家）	崔久嵬*	吉林大学第一医院
	陈克能	北京大学肿瘤医院
	陈明	中山大学附属肿瘤医院
	段建春*	中国医学科学院肿瘤医院
	董晓荣	华中科技大学协和医院
	冯继锋	江苏省肿瘤医院
	范云	浙江省肿瘤医院
	高树庚	中国医学科学院肿瘤医院
	韩宝惠	上海市胸科医院
	黄建安	苏州大学附属第一医院
	何勇	陆军特色医学中心
	胡毅	中国人民解放军总医院
	何雅億	同济大学附属上海市肺科医院
	李进	上海市东方医院
	柳菁菁	吉林省肿瘤医院
	李伟	蚌埠医学院第一附属医院
	卢铀	四川大学华西医院
	马智勇	河南省肿瘤医院
	潘宏铭	浙江大学医学院附属邵逸夫医院
	潘跃银	中国科学技术大学附属第一医院
	任胜祥*	同济大学附属上海市肺科医院
	苏春霞*	同济大学附属上海市肺科医院
	宋启斌	武汉大学人民医院
	宋勇	南京大学医学院附属金陵医院
	束永前	江苏省人民医院
	陶敏	苏州大学附属第一医院
	王长利	天津医科大学肿瘤医院
	王佳蕾	复旦大学附属肿瘤医院
	王绿化	中国医学科学院肿瘤医院深圳医院
	王哲海	山东省肿瘤医院
	王志杰*	中国医学科学院肿瘤医院
	王子平	北京大学肿瘤医院
	谢丛华	武汉大学中南医院
	许亚萍	同济大学附属上海市肺科医院
	杨帆*	北京大学人民医院
	杨农	湖南省肿瘤医院
	袁双虎	山东省肿瘤医院
	姚煜*	西安交通大学第一附属医院
	周承志*	广州医科大学附属第一医院
	周建英	浙江大学医学院附属第一医院
	朱广迎	中日友好医院
	朱波	陆军军医大学第二附属医院
	钟华	上海市胸科医院
	张力	中山大学附属肿瘤医院
	张兰军	中山大学附属肿瘤医院
	赵路军	天津医科大学肿瘤医院
	庄武	福建省肿瘤医院
